# The Effect of Single Accreditation on Medical Student Match Rates in Surgical Specialties

**DOI:** 10.7759/cureus.14301

**Published:** 2021-04-05

**Authors:** Ian Etheart, Stephanie M Krise, J B Burns, Kristen Conrad-Schnetz

**Affiliations:** 1 Surgery, West Virginia School of Osteopathic Medicine, Cleveland, USA; 2 Surgery, Ohio University Heritage College of Osteopathic Medicine, Cleveland, USA; 3 Trauma/Critical Care, East Tennessee State University, Johnson City, USA; 4 Surgery, South Pointe Hospital Cleveland Clinic Foundation, Cleveland, USA

**Keywords:** single accreditation, match, nrmp, aoa, surgical residency, surgical subspecialties

## Abstract

Introduction

The year 2020 marked the first year in which a match under single accreditation took place. Both osteopathic (DO) and allopathic (MD) students would participate in the first match cycle without a dedicated DO match system. Our primary objective was to investigate how single accreditation has impacted the DO applicants attempting to match into surgical specialties. Our secondary objective was to investigate the impact of single accreditation at the program director (PD) level and whether or not this process would see a change in DO PD distribution in previously American Osteopathic Association (AOA)-approved programs.

Method

Information on number of applicants and post-match positions was gathered from AOA and National Residency Match Program (NRMP) websites. Credentials of PDs were obtained from the Accreditation Council on Graduate Medical Education website. Based on the available data, the following surgical specialties were compared for the years 2020, 2018, and 2016: General Surgery, Neurological Surgery (NSGY), Orthopedic Surgery, Otolaryngology/ENT (ENT), Plastic Surgery, and Thoracic Surgery. Data from 2016 were not included in the results as the AOA match results analysis was insufficient and unable to be directly compared to the NRMP data. Results of matched DO and MD applicants were compared using bivariate analysis. A p-value of <0.05 was considered significant.

Results

From the year 2018 to 2020, the DO applicants saw a decrease of 3% in the total number of matched postgraduate year 1 spots in surgical specialties. NRMP results from 2020 saw that 51.7% of DO applicants matched and 67.7% (p < 0.001) of MD applicants matched for the specialties examined. Percent of matched:applied for DO applicants was lower than MD applicants in the fields of NSGY (p < 0.001), ENT (p < 0.001), Plastic Surgery (p < 0.001), General Surgery (p < 0.001), and Thoracic Surgery (p = 0.011). After evaluating 60 former AOA General Surgery programs, 56% were found to have MD as PD. Another 26 former AOA surgical programs were investigated, and 58% were found to have MD PD.

Conclusion

Single accreditation has impacted the match process now that a large number of both MD and DO applicants are using the NRMP match system for postgraduate placement. Based on the available data, our results indicate that in the examined surgical specialties, there is a statistically significant difference in the number of MD and DO residents.

## Introduction

Medical students now face an uncertain future with single accreditation and a single match process. The year 2020 would be the first year in which the National Residency Match Program (NRMP) would be the main program for matching into Accreditation Council on Graduate Medical Education (ACGME)-approved residency programs. This eliminated the match system delivered through the American Osteopathic Association (AOA), which offered DO students residency positions at traditional DO institutions. Prior to 2020, DO applicants could choose to apply through the AOA match or through the NRMP [1]. Allopathic students applied and matched primarily utilizing the NRMP.

The move to a single accreditation has resulted in the closure of many traditional AOA programs, including surgical residency programs [1]. Some programs that completed full transition to ACGME accreditation currently have an MD as PD [2].

It has not yet been demonstrated if and how the transition of previously AOA-approved programs to ACGME-approved programs has affected the match rates of MD and DO medical students. The purpose of our investigation was to see what effects a single accreditation system has on the match results of MD and DO medical students in surgical specialties. Currently, there is a paucity of data examining the effects. We hypothesize that single accreditation has diminished opportunity to match into surgical specialties for DO applicants while remaining relatively unchanged for MD applicants. 

## Materials and methods

Data were collected from NRMP, AOA, Association of American Medical Colleges (AAMC), ACGME, and individual program websites. Data from the NRMP and AOA were used to examine the amount of postgraduate year 1 (PGY-1) positions that were given to MD and DO applicants. The groups compared were MD and DO applicants in their senior year of undergraduate medical education. Based on the available data, the following were the surgical specialties for the year 2020 and 2018: General Surgery, NSGY, Orthopedic Surgery, ENT, Plastic Surgery, and Thoracic Surgery.

The number of applicants was collected from the AAMC website. To assess the effects on the MD applicants, data from the NRMP were utilized. To assess the effects on the DO applicants, the data were combined from AOA and NRMP prior to the year 2020. For the year 2020, the data published in the 2020 Main Residency Match Report, provided by the NRMP, were used for both DO and MD applicants. Additional information for the number of DO applicants for the year 2018 was found using the NRMP Supplemental Outcomes data.

A bivariate analysis using chi-square test and Fisher's exact test was performed in order to evaluate the percent difference of matched:applied for both MD and DO applicants. SAS 9.4 software (SAS Institute Inc., NC, USA) was used for analysis. A p-value of <0.05 was considered statistically significant.

To examine program leadership, previous AOA General Surgery Residency Programs were reviewed using the ACGME website. Assessment of credentials of PD, either MD or DO, was done using the program information listed on the ACGME website. The same method was used for other traditional AOA and included fields of Urological Surgery, NSGY, and Plastic and Reconstructive Surgery. 

## Results

**Figure 1:**
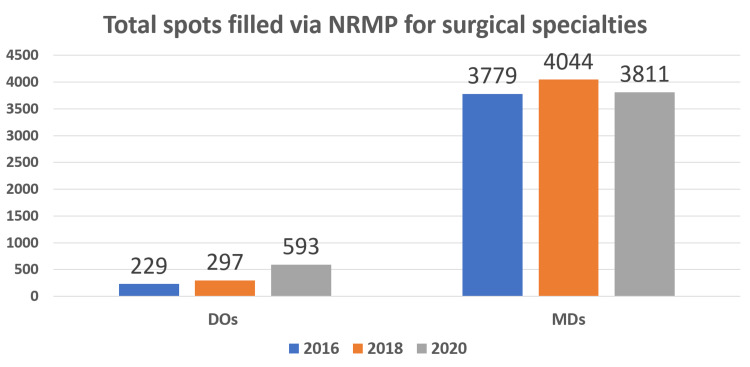
Number of surgical specialty postgraduate year 1 spots occupied by DO and MD applicants in 2016, 2018, and 2020.

The total number of matched spots for the DO applicants in 2016 was 229 for the AOA match and 213 for the NRMP match, a total of 442. For the year 2018, the total number of DO matched spots was 297 for the AOA match and 384 for the NRMP match, a total of 613. In the 2020 combined match system, 593 matched spots were occupied by the DO applicants. For those same years, the number of matched spots for the MD applicants was 3379, 4044, and 3811, respectively (Figure 1).

**Figure 2:**
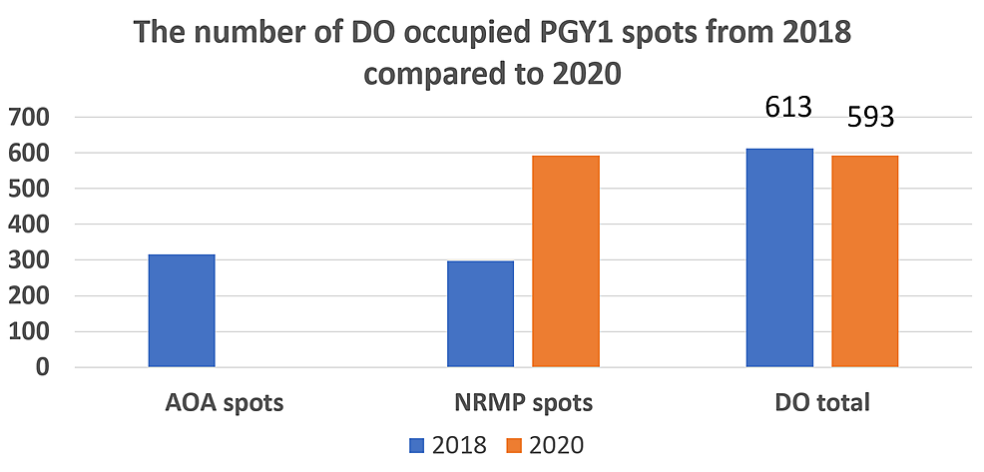
In 2018, the AOA match system was available for DO applicants attempting to match into surgical specialties. In 2020, with the AOA match system no longer available, the number of DO applicants occupying postgraduate year 1 surgical specialty spots declined by 3%. AOA, American Osteopathic Association; NRMP, National Residency Match Program

From the 2018 match to the 2020 match, the total number of PGY1 spots occupied by DOs decreased from 613 to 593, a decrease of 3% (Figure 2).

**Figure 3:**
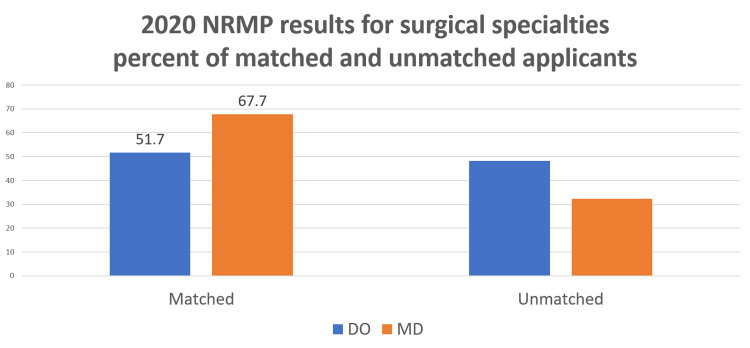
At the conclusion of the 2020 match, 51.7% of DO applicants and 67.7% of MD applicants matched into surgical specialties (p < 0.001).

In 2020, the percent matched for the DO applicants was 51.7% compared to 67.7% for the MD applicants (p < 0.001) (Figure 3).

**Figure 4:**
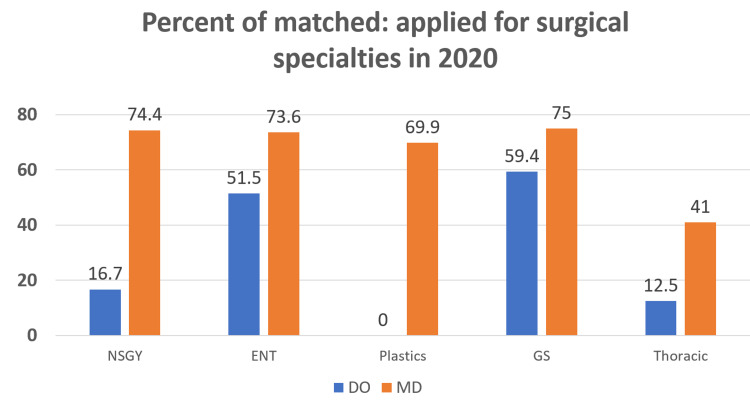
Percent of matched:applied for postgraduate year 1 surgical specialties in 2020. NSGY, Neurological Surgery; ENT, Otolaryngology/ENT; GS, General Surgery

The match percent for five surgical specialties in 2020 was also examined between the DO applicants and the MD applicants. This was calculated with the total number of applicants matched divided by the total number of applicants. For NSGY, 16.7% of the DO applicants were matched and 74.4% of the MD applicants were matched (p < 0.001). For ENT, 51.5% of the DO applicants were matched and 73.6% of the MD applicants were matched (p < 0.001). The subspecialty of Plastic Surgery did not match any DO applicants to a PGY-1 spot in 2020, but 69.6% of the MD applicants were matched (p < 0.001). General Surgery was the surgical subspecialty that matched the greatest proportion of DO applicants, 59.4% DO applicants and 75% MD applicants. This was the largest DO concentration in PGY-1 surgical specialty spots but was still found to be a smaller percentage compared to the MD applicants (p < 0.001). In Thoracic Surgery, 12.5% of the DO applicants were matched and 41% of the MD applicants were matched (p = 0.011) (Figure 4).

**Figure 5:**
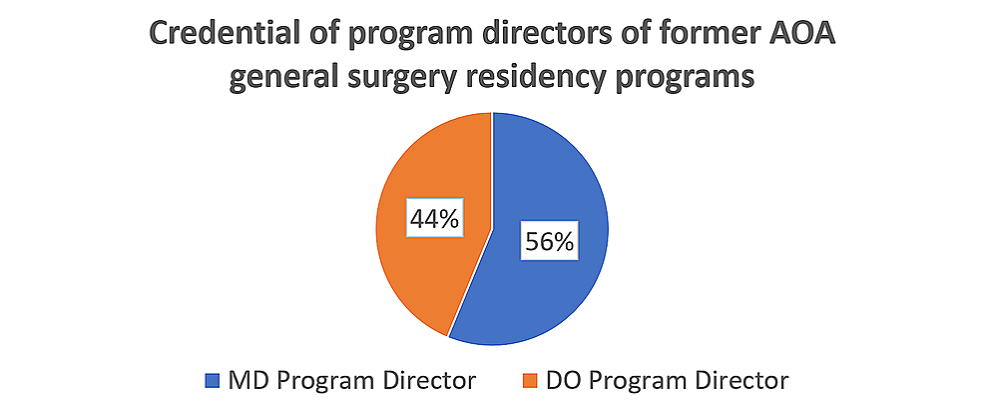
An investigation of 48 former AOA General Surgery residency programs revealed that 27 had an MD listed as PD and 21 had a DO listed as PD on the Accreditation Council on Graduate Medical Education website. AOA, American Osteopathic Association; PD, program director

Sixty former AOA GS residency programs were examined. Twelve programs closed or did not make transition to full ACGME accreditation. The remaining 48 were searched on the ACGME website. Twenty-seven programs had an MD listed as the PD and 21 had a DO listed as PD (Figure 5).

**Figure 6:**
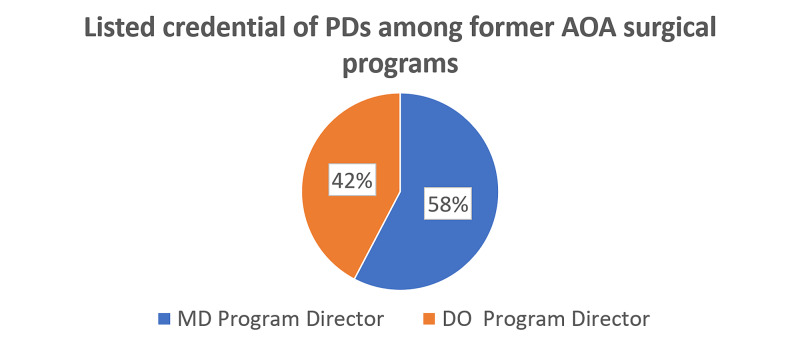
A group of 44 former AOA surgical residency and fellowship programs were also investigated to determine the credentials of the PD. This group included neurological surgery residencies, urological surgery residencies, and surgery fellowships. Of the 15 programs that made the transition to full Accreditation Council on Graduate Medical Education accreditation, 15 had an MD listed as PD and 11 had a DO listed as PD. AOA, American Osteopathic Association; PD, program director

The investigation was expanded to include 44 additional surgical programs. This group was made up of surgery fellowships, NSGY residencies, and urological surgery residencies. Eighteen of those programs did not transition to full ACGME accreditation or closed. Of those remaining, 15 were found to have an MD listed as the PD and 11 were found to have a DO listed as the PD (Figure 6).

## Discussion

This review of the available match data demonstrates that the first year under a single accreditation saw a lower percentage of DO applicants matching into surgical specialties compared to MD applicants. While DO applicants may occupy more PGY-1 spots in the NRMP match than they did in previous years, overall, there are fewer DO applicants matching to PGY-1 spots of the surgical specialties examined. The percent of MD applicants matched and the number of MD applicants in PGY-1 spots for the same surgical specialties have remained relatively stable. The elimination of the AOA match system has potentially reduced opportunities for the DO applicants wishing to pursue careers in GS or specific surgical specialties. Craig et al. found that the DO applicants have difficulty matching into competitive residency programs under single accreditation [3]. Plastic Surgery is just one specialty that the DO applicants have struggled to match into. The analysis of DO representation in competitive specialties (Dermatology, Otolaryngology, Orthopedic Surgery, Neurosurgery, and Plastic Surgery) by Craig et al. found that DO representation in these fields increased from 0.5% to only 0.6% under single accreditation. Additionally, for former AOA positions in these specialties, 27.6% were occupied by the MD applicants [3]. At the surgical specialty level this discrepancy was most pronounced in the fields of Plastic Surgery and Thoracic Surgery. In the 2020 NRMP, 0 DO applicants matched into a PGY-1 Plastic Surgery position; however, three US international medical graduate (IMG) and six non-US IMG applicants matched into Plastic Surgery. There was a single DO graduate who matched into Plastic Surgery residency, but not as a PGY-1 [4]. Despite a total of 291 applicants applying for 18 spots, of the total applicants only 13 were DO students. While applicants in the NRMP system are free to rank any program they choose, often applicants choose to rank programs who have interviewed them. What is unknown from our data gathering is how many DO applicants submitted applications to ACGME Plastic Surgery residency programs during the 2020 cycle but were not offered interviews. These areas may prove to be an integral step when looking at ways to preserve the tradition of osteopathic physicians in surgery - educating programs and their leadership about osteopathic applicants. This could apply even to former AOA programs. As reported earlier, 58% of former AOA GS residency programs have an MD listed as the PD. Data gathered from future matches will show if this statistic has adversely affected osteopathic applicants wishing to pursue careers in surgery.

The main limitation of this study was lack of available data. The NRMP provides a post-match report that includes information on the number of applicants for each specialty [5-7]. The AOA data did not provide any information on the number of applicants for a given specialty but only listed the total number of spots occupied and unoccupied after the match [8,9]. While the NRMP is the main match for ACGME residencies, it is not the only match available for DO students. The Urological Surgery Match, San Francisco Match, and the Military Match are just three examples. We did not have data available for these or other match systems. Data from those match systems were not included in our analysis. It is possible that the DO applicants were able to gain surgical residencies from these other matches and incorporation of such information with this project will give a better sense of the impact of single accreditation on DO applicants.

In the future, more data will need to be gathered from subsequent matches to trend how DO students will match into surgical residencies under a single accreditation system. Along with trending matches overall, additional data can be gathered from other specialties to assess how single accreditation has impacted those fields. In doing so, utilization of American College of Osteopathic Surgeons' (ACOS') resources will be an important step in the process. Such actions could lead to the creation of new, ACGME-approved, DO-led residency programs that will offer DO applicants additional opportunities to train. Smaller programs that were unsure or unable to make transition to ACGME accreditation may wish to re-establish training programs in the future. Some efforts are already underway by ACOS. Currently, the ACOS student section has implemented a project to identify residencies where DOs have historically matched well as well as a survey of PDs. Work at the administrative level in residency programs will allow for DO applications to be viewed equally with MD applications and increase the pool of qualified applicants available to a given program. Finally, the creation of a Graduate Medical Education Subcommittee will work to design local, regional, and national strategies to address this issue going forward.

## Conclusions

As a result of single accreditation, the elimination of the AOA match system and the increased utilization of the NRMP for residency matching have led to a change in outcomes for the DO applicants. Compared to MD applicants, the DO applicants are at a disadvantage when it comes to the surgical specialties that we investigated. General Surgery was an area where the DO applicants matched well relative to other surgical subspecialties such as Plastic Surgery, NSGY, and Thoracic Surgery. For the 2020 match, no DO was able to obtain a PGY-1 spot in Plastic Surgery. While there is only a single year of data available, we hope to use this opportunity to inform both applicants and programs about how single accreditation has impacted the osteopathic contribution in the field of surgery. Future data-gathering combined with advocacy by ACOS and others will work to preserve the tradition of osteopathic surgery and ensure that the future DO students have opportunities to get trained in their desired field.
